# Can Economic Growth and Environmental Protection Achieve a “Win–Win” Situation? Empirical Evidence from China

**DOI:** 10.3390/ijerph19169851

**Published:** 2022-08-10

**Authors:** Zhen Yang, Weijun Gao, Jiawei Li

**Affiliations:** 1 College of Civil Engineering and Architecture, Weifang University, Weifang 261061, China; 2 Innovation Center for CIM + Urban Regeneration, Qingdao University of Technology, Qingdao 266033, China; 3 Faculty of Environmental Engineering, The University of Kitakyushu, Kitakyushu 808-0135, Japan; 4 Innovation Institute for Sustainable Maritime Architecture Research and Technology (iSMART), Qingdao University of Technology, Qingdao 266033, China

**Keywords:** economic performance, environmental pressure index, nighttime light, decoupling analysis, sustainable development

## Abstract

Achieving a “win–win” situation regarding economic growth and environmental protection has become a common goal for sustainable development in all countries around the world. As the world’s largest developing country and the second largest economy, China has been striving to maintain economic growth while improving environmental quality to achieve its sustainable development goals. Applying the decoupling approach, a model widely used to quantify the relationship between the environment and the economy, this study analyzed the relationship between the economy and the environment, examining the decoupling performance of economic growth and environmental impacts in 30 Chinese provinces, autonomous regions, and municipalities to investigate whether economic growth and environmental protection have achieved a “win–win” situation. Nighttime light (NTL) data were used to measure the performance of economic growth. In addition, an environmental pressure index (EPI) assessment framework covering 6 primary and 11 secondary indicators was constructed to measure the environmental quality of China over time. First, NTL data proved to be a valid data source for assessing decoupling performance; second, environmental pressure at both the national and provincial levels significantly decreased during the study period; third, the relationship between the economy and the environment has been further improved, and economic growth and environmental protection have achieved a “win–win” situation. These findings offer an in-depth analysis of the decoupling of the economy and the environment in China and serve as a guide for future implementation strategies for sustainable development in various regions.

## 1. Introduction

At present, the world’s countries are aware of the crucial importance of reducing environmental pollution while maintaining economic prosperity. That is to say, decoupling environmental pollution from economic growth (EG) in order to achieve sustainable development has become a widespread objective [1]. China’s economic growth has achieved incredible things since the reform and opening up, but it has also been accompanied by major environmental pollution, including dust, water pollution, solid waste, and pollutants other than the common forms such as sulfur dioxide and nitrogen oxides [2,3,4,5,6,7,8]. This is not only the most serious challenge to the survival and development of natural ecosystems and human beings, but it also causes huge economic losses [9,10]. According to pertinent data, environmental pollution costed China’s economy 3.05% of its GDP between 2004 and 2012 [11]; however, in 2019 this figure exceeded 6%, and in 2021, the World Bank, the Chinese Academy of Sciences, and the Environmental Protection Agency estimated this proportion to be a staggering 10%. Therefore, there is an urgent need to achieve a “balance” between the economy and the environment at the national level; therefore, while maintaining economic growth in size, the pursuit of environmental pollution at the lowest cost is also an inevitable choice for achieving sustainable regional development.

To address the growing environmental problems, the central and provincial governments have developed several action plans and targets in recent years to control the urgency of environmental issues, namely air, water, and soil pollution, as well as carbon emissions. For example, the state has mandated that 93% of urban water supplies must meet “drinking water” standards by 2020 [12], strict air quality targets have been set in regions such as the Yangtze River Delta and the Pearl River Delta [12], there has been an implementation of documents on environmental taxes and cap-and-trade policies based on market policies [13,14,15,16], China has committed to carbon neutrality and carbon peaking targets [17,18,19], etc. The implementation of the above plans and actions will help us to comprehensively address the serious environmental problems that China faces with respect to its economic growth. Nevertheless, China is still at the center of the worldwide debate on environmental issues. The possible reasons for this are, on the one hand, differences in the environmental indicators discussed amongst scholars, i.e., more irreversible environmental phenomena are discussed, such as soil erosion, land desertification, arable land reduction, soil quality degradation, etc., and on the other hand, a bias in the population’s perception of environmental risks, i.e., areas with higher perception of environmental risks, instead their environmental problems are less prominent and even more efficiently managed. Therefore, the pursuit of decoupling between EG and environmental damage is a difficult medium- to long-term task.

However, China’s environmental problems are far from being fundamentally solved. As a fast-growing economy and the largest developing country, China is under tremendous pressure to pursue the decoupling of EG and environmental damage [11]. On the one hand, China is seeking sustained economic growth in keeping with its ambitious urbanization mission, while on the other hand, a sloppy developmental approach and a high-emission energy structure are further degrading the environment [19,20,21]. Currently, ecosystems are being destroyed and restrictions on resources are being tightened [22]. One explanation is that the “stock effect” is beginning to emerge due to China’s massive pollution emissions, which may exceed its ecological carrying capacity. China’s environmental issues have become more severe and have given rise to several societal issues, particularly in recent years. So, China must alter its current economic growth trajectory in order to achieve sustainable development. Building a green manufacturing process and achieving green economic growth are the objectives of changing the economic growth mode from an environmental standpoint [23]. In fact, at the 18th National Congress, the central government put forward a vision of harmonious economic and environmental development and characterized greening as a “political mission” of the Chinese government [24]. Therefore, the development of a “win–win” situation with respect to economic growth and environmental protection is an important strategy at the national level. In addition, different economic, social, and environmental characteristics are present in different areas of China, and the economic performance and environmental conditions of different regions vary greatly. Analyses of the decoupling of these two factors in each area are required, so that emission reduction policies can be tailored to different regions to reduce environmental pressure and achieve sustainable development.

Achieving a “win–win” situation for economic growth and environmental protection requires that the needs of economic development be met with minimal environmental costs, or in other words, decoupling economic development from environmental damage. However, a proper understanding of the decoupling of economic development and environmental damage in different regions depends on the validity of the data and the comprehensiveness of the indicators [17,25]. In other words, conducting a decoupling analysis requires accurate data sources and a comprehensive evaluation system. Therefore, the main motivations of this research are as follows: (1) to understand the urgency of environmental protection in different regions of China, and (2) to correctly examine the evolutionary characteristics of the decoupling state between economic growth and environmental damage in China over the years in order to tailor policy measures for different regions on the one hand, and to provide China’s experience in environmental sustainability development on the other. Based on the above analysis, this study will investigate the decoupling status of 30 provinces, autonomous regions, and municipalities in China using nighttime light data and a comprehensive environmental pressure index to improve the accuracy of the data and to tailor reasonable emission reduction measures to different regions.

The following material defines the structure of this paper: A literature review is conducted in Section 2, and the study methodology, research data, and research steps are presented in Section 3. The findings and analyses between environmental sustainability and economic growth at different levels in China from 2004 to 2019 are shown in Section 4, Section 5 provides a discussion of the decoupling performance of regional economies and the environment, and Section 6 presents the conclusions and policy implications.

## 2. Literature Review

The non-monotonic link between different pollutants and income has recently drawn a great deal of interest from academics and has been thoroughly examined [26,27,28,29,30,31,32]. This idea was first proposed by Grossman and Krueger in their analysis of the North American Free Trade Agreement (NAFTA) [33]. They concluded that although economic development causes environmental quality to decline, it also causes environmental degradation to rise. In other words, there could be a tipping point in the relationship between environmental performance and economic development. This theory is widely known as the Environmental Kuznets Curve (EKC) hypothesis. In this context, a number of contradictory insights have emerged from the research. For example, Ahmeda and Long find that the concept of an inverted U-shaped curve is correct only in the long term [31], while Zhao uses the growth rate of gas emissions to reach the opposite conclusion, observing that the EKC hypothesis is widely supported in the short run, but not evident in the long run [34]. Meanwhile, other studies have proposed different shapes of curves to describe the relationship between economic growth and environmental impacts. Lopez and Mitra’s hypothesis is a U-shaped curve instead of an inverse curve. In contrast to the EKC hypothesis, this implies an initial high rate of pollution, followed by a decrease in pollution until a specific level of economic growth is reached, and then another increase [32]. Interestingly, the N-shaped curve is similarly captured in the analysis of the relationship between the two, where continued economic growth is accompanied by ecological damage that once again begins to increase [35,36,37,38,39]. Table 1 summarizes the findings of studies on the relationship between the environment and economy and their authors. In conclusion, the EKC theory has yielded a variety of findings, and the main reason for this phenomenon is the choice of variables. Lopez and Mitra consider the model to have been applied adopting inadequate environmental variable [32]; Cleveland warns that a single variable may introduce bias, as manufacturing processes produce several forms of pollutants [40]; Brajer believes that current pollutants should involve multiple variables and that measuring environmental impacts based on a single variable would be a difficult task [41]; Brajer and Tevie believe that different variables should be used to measure environmental impacts [41,42]. In general, univariates provide a short-sighted approach to solving practical problems related to the overall impact of ecosystems.

Decoupling theory has been extensively employed to examine the link between economic development and environmental deterioration as research has advanced in recent years [54,55,56,57,58,59]. Decoupling refers to severing the connection between commercial products and environmental concerns or energy use [60]. It is one of the measurement techniques now used by institutions at the regional and national levels. Studies based on decoupling emphasize the separation of energy use and pollution emissions (e.g., carbon dioxide, sulfur dioxide, dust, solid waste, and wastewater). The former refers to a decline in the levels of emissions produced per unit of economic production, whereas the latter refers to a decline in overall emissions as GDP increases. For example, Song used a decoupling index to compare differences in the decoupling trends with respect to the GDP and CO_2_ emissions between developed and developing countries [61]. Wu and Shan et al. presented a study on the decoupling of GDP and CO_2_ at the regional level in China [19,62]. Wang focuses on the evolutionary trends of GDP growth and SO_2_ decoupling at the provincial level in China [63]. Other single variables are similarly used as indicators of environmental impact, including threatened species, water pollution, forest destruction, conversion of agricultural land, and industrial waste.

However, the current analyses of economic growth and environmental impacts based on decoupling theory have two holes in their research. First, when performing decoupling analysis, GDP, which is readily obtainable from statistics yearbooks, is the most common indicator used to measure the economic performance of a region or city. However, the accuracy of statistics yearbooks is limited. Previous research has demonstrated that our statistical yearbooks include some accuracy, particularly when it comes to the distinction between bias in the data collection and data calculation [64]. Recently, several researchers have attempted to employ remote sensing techniques to investigate regional economic growth in order to increase the accuracy of the data, such as nighttime light (NTL) data [65,66,67]. For example, using NTL data, Qin discusses the economic development of Chinese cities and examines how urban expansion affects that development [68]. Wang demonstrates that the influence of human variables on statistical inaccuracies brought on by human behavior in the measuring of economic development can be eliminated using NTL data [69].

Second, there has not been enough research conducted in the past to examine how these changes in the decoupling levels are integrated into the creation of policies. Existing studies mostly use single pollutants as indicator variables for environmental impacts, such as PM_2.5_, CO_2_, NO_x_, SO_2_, wastewater, etc., which emphasize a specific source of environmental pressure and do not enable a comprehensive quantification of environmental impacts or environmental performance [1,13,59,62,70,71,72]. One pollutant may be decreased as a result of a single source of attention, while others may see an increase. By not addressing reduction targets as a whole, research using this strategy often concludes that the main solution is to apply expensive end-of-pipe (EOP) treatments for each pollutant. Some scholars have criticized studies that use a single pollutant as an environmental indicator and have advocated the use of an environmental composite index to develop the analysis, since a composite indicator can consider more information and can capture all the impacts on various environmental dimensions, thus covering a richer and broader range of ecosystem characteristics [30,73,74,75]. Therefore, building comprehensive indicators that may be used to study the decoupling connections and can function as proxies for environmental consequences or environmental deterioration between economic development and environmental repercussions can offer additional and more thorough insights and introduce fresh views to strengthen the conversation on how the economy and the environment interact, which will lead to better decision making.

By addressing these gaps in the existing literature, this study adopts NTL data to measure China’s economic performance at the provincial level, while constructing a multidimensional index system of regional environmental pressure indices (EPI) to analyze the decoupling between economic development and environmental impacts in 30 Chinese provinces, autonomous regions, and municipalities from 2004 to 2019, and to investigate whether China’s economic growth has achieved a “win–win” situation with environmental protection.

## 3. Methods

**Figure 1:**
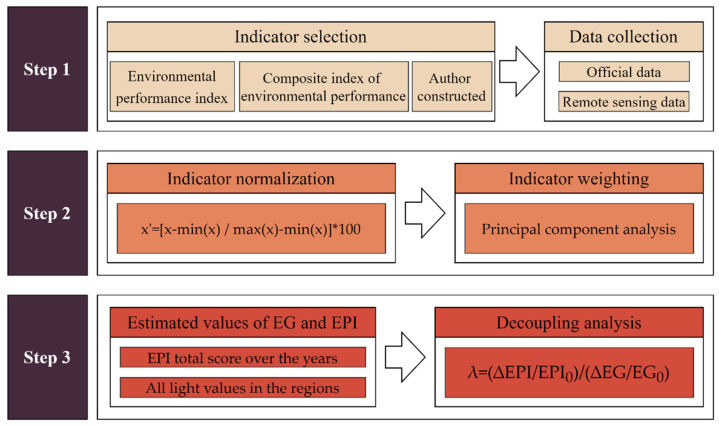
Three interrelated steps were involved in the assessment and analysis of the economic and environmental decoupling performance in China.

Three interrelated steps (Figure 1) were involved in the investigation of the decoupling between economic growth and environmental impacts at two levels (national and provincial) in China.

**Step 1. Indicator selection and data acquisition.**

Despite the greater availability of data nowadays, the selection of indicators should adhere to fundamental standards, with relevance, completeness, reliability, and accessibility being the most important considerations [76]. Among these, the environmental performance index—jointly customized by the Yale Center for Environmental Law and Policy at Yale University and the International Geoscience Information Network Center at Columbia University—is the best known [77]. In accordance with the two policy areas of environmental health and ecosystem vitality, the most recent evaluation framework groups environmental indicators according to natural resources, ecological conditions, and environmental pollutants. Another global environmental performance scoring system is the comprehensive index of environmental performance (CIEP) proposed by Spanish scholars [78]. The CIEP was developed from Drivers–Pressure–State–Exposure–Effectiveness–Action and supplies a summary of 19 indicators that are scored annually for 152 countries.

Nevertheless, not all of these metrics are relevant to China’s environmental issues. The components of the composite indicator, on the one hand, must accurately reflect the environmental challenges that China has encountered throughout time, and on the other, the time-scale data must originate from reputable and authoritative sources. Therefore, there are limitations in the quantitative studies of environmental pressure indices at the provincial level in China. Zuo et al. constructed an environmental performance assessment framework consisting of 39 indicators covering environmental health, ecological protection, sustainable resource use, and environmental management to evaluate and rank the overall environmental performance of 30 Chinese provinces, autonomous regions, and municipalities during the 11th Five-Year Plan period [79]. However, the large number of indicators adds to the difficulty of tracking and updating changes over time. In this context, we developed a new assessment framework that represents the state of environmental pressure in China’s 30 provinces, autonomous regions, and municipalities by covering six dimensions measured by 11 environmental indicators. Table 2 summarizes the relevant indicators of the constructed environmental pressure index, with each level describing a particular dimension of the environmental conditions. Specifically, provincial PM_2.5_ is used as an environmental measure of air quality, one of the most often utilized indicators; greenhouse gases are represented by carbon dioxide emissions; sulfur dioxide, nitrogen oxides, and dust are used to represent waste gases; industrial wastewater and chemical oxygen demand are used to represent wastewater; hazardous waste and general industrial solid waste are used to represent solid waste; and total urban environmental infrastructure investment and total industrial pollution treatment completion were chosen as proxies for environmental resilience because the use of pollutant discharge fee (PDF)-defined monetization instruments enables the conversion of pollutant emissions into economic measures to control environmental problems [10].

In this paper, NTL data are utilized to access economic performances at different levels in China. The data consist of a dataset published in the Earth System Science Data Journal and shared in the Harvard Dataverse by Chen et al. [80]. This dataset’s information can be a great resource for a great deal of research on human activity, including research analyzing urban dynamics and economic development. The carbon dioxide dataset in this study was obtained from carbon emission accounts and datasets (CEADs https://www.ceads.net.cn, accessed on 10 July 2022), which has been a widely used source in carbon research and has achieved fruitful results. Other environment-related indicators were gathered from the China Statistical Yearbook, China Environment Yearbook, and the China Urban Construction Yearbook.

**Step 2. Indicator processing and aggregation.**

After organizing the environmental indicators at the two levels in China, we dimensionless sized all indicators as follows:

(1) $$x^{\prime }=x-\min\left(x\right)/\max\left(x\right)-\min\left(x\right)\;\times \;100$$

where $x^{\prime }$ denotes the normalized value, x denotes the initial indicator value, and min and max denote the indicator’s lowest and upper bounds, respectively, for the best and worst performances. The highest limit of all normalized values is set to 100, while the lower limit is set to 0. The rescaled metrics are denoted as ascending metrics, with higher values indicating higher environmental pressure.

**Figure 2:**
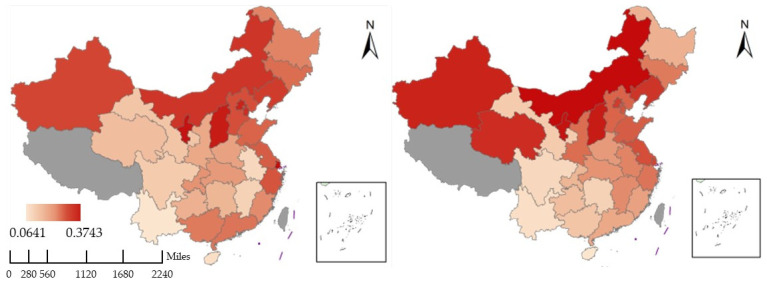
Calculated EPI in 2004 (**left**) and 2019 (**right**) for 30 Chinese provinces, autonomous regions, and municipalities.

Setting up weighted criteria is crucial for scientific evaluation. We decided to use the principal component analysis (PCA) approach to figure out the weighting factors for each indicator in order to avoid placing an excessive amount of focus or effect on individual subjectivity. PCA is mostly utilized as a statistical method for developing predictive models and exploratory data analysis. By computing a data covariance (or correlation) matrix, an analysis can take into account the physical and causal links between variables and genetic distances. Figure 2 depicts the calculated EPI for 30 Chinese provinces, autonomous regions, and municipalities.

**Figure 3:**
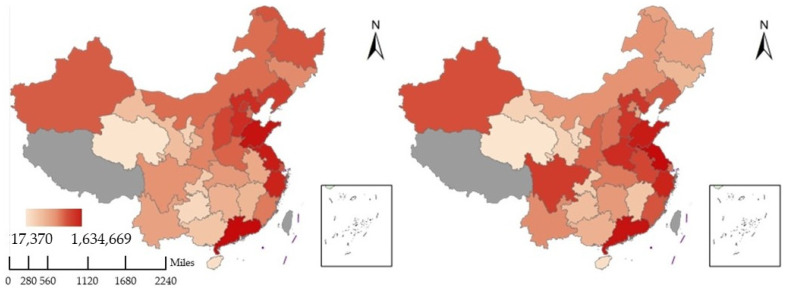
Calculated EG in 2004 (**left**) and 2019 (**right**) for 30 Chinese provinces, autonomous regions, and municipalities.

As a proxy variable for economic performance, NTL data also need to be processed. The NTL data available in the Harvard Dataverse are at the global level. Therefore, data related to each province in China needed to be retrieved. The retrieval process consisted of two steps: firstly, the original dataset needed to be imported into Geographic Information System (GIS), and the corresponding NTL ranges were extracted according to the provincial map masks of China from 2004–2019. Figure 3 shows the NTL data for 2004, 2007, 2010, 2013, 2016, and 2019. Then, the NTL data were extracted according to the mask of China, and the light values (the unit of the nighttime light data is DN, the higher the value, the more intense the light, i.e., the higher the level of economic development, and vice versa) of different regions in different years were captured by partitioning statistics in the GIS. Due to the enormous sample size, Table 3 only displays the NTL data values for the ten sample provinces throughout the research period.

**Step 3. Estimated values of EG and EPI over time and decoupling analysis.**

After determining the weighting coefficients for each indicator using the PCA method, we aggregated the total environmental pressure scores for the assessment framework covering 11 indicators. In addition, based on the administrative boundaries of each province, we similarly aggregated the sum of all light values within the administrative boundaries of different provinces as the performance of regional economic development. A total of 16 national scores and 480 provincial scores were obtained as a consequence of the aggregation, and they were all utilized to study the spatial and temporal dynamics of the “win–win” condition of economic growth and environmental protection in China at various levels throughout the study’s time.

Decoupling models were used to analyze whether a “win–win” situation of economic growth and environmental protection has been achieved at different levels in China. To date, two decoupling models have been widely applied, namely, the OECD model [60] and the Tapio model [81]. Compared to the former, the Tapio model provides a finer distinction between decoupling states and explains the decoupling effects based on a flexible incremental analysis of dynamic data. Instead of evaluating the absolute values of variables, the model uses the idea of elasticity to quantify sensitivity to incremental values. Therefore, the Tapio model was selected to test the decoupling states between economic growth and environmental pressure, as shown in Equation (2):

(2) $$\lambda =\frac{\Delta EPI/EPI_{0}}{\Delta EG/EG_{0}}=\frac{\left(EPI_{t}-EPI_{0}\right)/EPI_{0}}{\left(EG_{t}-EG_{0}\right)/EG_{0}}$$

**Figure 4:**
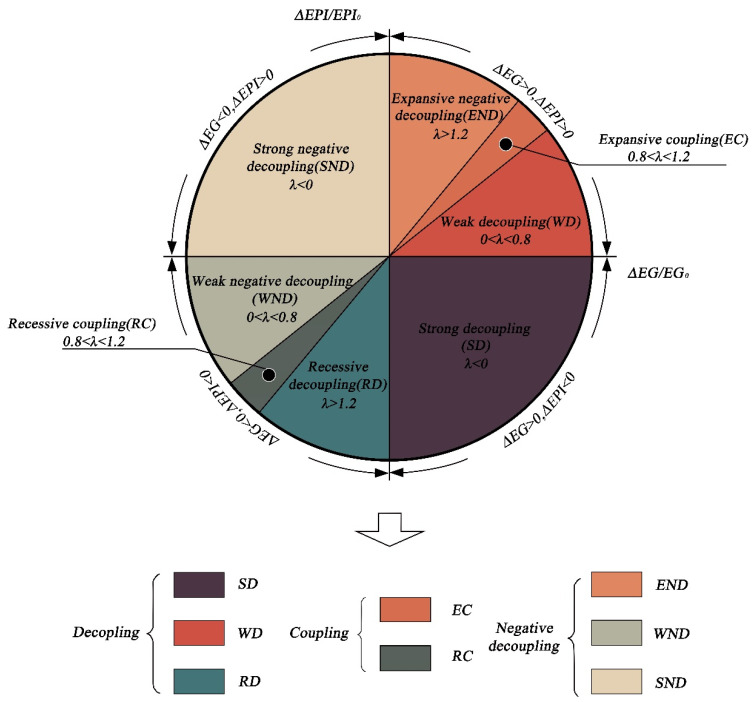
Classification of decoupling states in the Tapio model.

In Equation (2), ΔEG and ΔEPI denote the changes in economic performance and environmental pressure indices between the start year (0) and the end year (t), respectively. The model provides intervals of changes in elasticity values of about 1.0, plus or minus 20%, to precisely differentiate the decoupling states, preventing tiny changes from being mistaken for substantial ones. The decoupling states corresponding to λ are shown in Figure 4 and are divided into three major categories and eight subcategories.

i. Decoupling (D). The first major category is the decoupling state, which contains three subcategories and usually refers to a situation where the environmental pressure is rising lower than economic growth. This indicates that the dependence of economic growth on environmental damage as well as resource consumption are weakening. Weak decoupling (WD) indicates that both are increasing, but that economic growth is significantly faster than environmental pressure. Recessive decoupling (RD) indicates that both are decreasing, but environmental pressure is decreasing faster, and strong decoupling (SD) indicates that environmental pressure is decreasing while the economy is growing. Therefore, in this paper, we define the SD state as the “win–win” state between the EG and EPI.
ii. Coupling (C). The second major category is the coupling state, which contains two subcategories and usually refers to the fact that the changes in environmental pressure and economic performance show almost the same rate of change. This indicates a strong dependence between economic development and environmental damage. In this case, recessive coupling (RC) indicates that both fall at similar rates and expansive coupling (EC) indicates that both rise at similar rates.
iii. Negative decoupling (ND). The third major category is the negative coupling state, which contains three subcategories and usually refers to a situation where environmental pressure is significantly greater than economic growth, which is a completely unsustainable state. Among them, expansive negative decoupling (END) indicates that both are increasing with a more pronounced rise in environmental pressure, strong negative decoupling (SND) indicates an increase in environmental pressure and an economic recession, and weak negative decoupling (WND) indicates that both are decreasing, but with a more pronounced economic recession.

## 4. Results and Analysis

### 4.1. Results of Decoupling Status between Economic Growth and Environmental Pressures at the National and Provincial Levels

**Figure 5:**
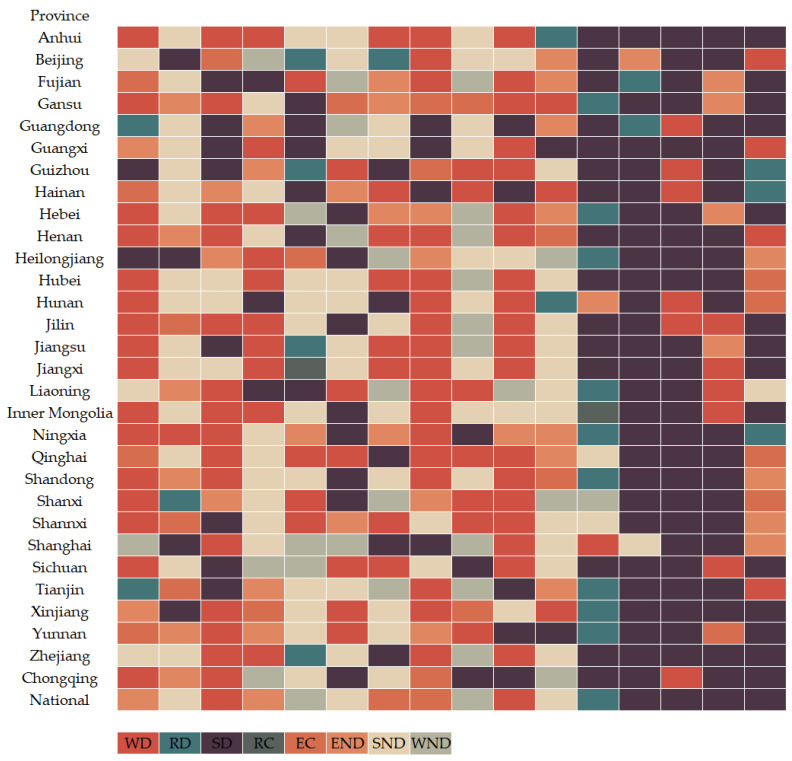
Decoupling status of nations and provinces from 2004 to 2019 (the first column is 2004, and so on, the last column is 2019).

By adopting the data mentioned in the previous section and providing it to the analytical model, it is possible to obtain the change in economic growth, the environmental pressure index, and the elasticity coefficient λ at the two levels for the period from 2004–2019. Table 4 presents the values of Δ*EPI*, Δ*EG* and λ for the sample city Beijing during the time period studied. Figure 5 summarizes the decoupling status at the national and provincial levels for the period 2004–2019.

### 4.2. Analysis of the Temporal Evolution of the Decoupling between Environmental Pressure and Economic Performance

The patterns in the temporal evolution of the decoupling state between economic performance and environmental pressures at two levels—the national level and the provincial level—were examined in this section.

#### 4.2.1. Decoupling between Environmental Pressure and Economic Performance at the National Level

Figure 5 summarizes the trends in the temporal evolution of the decoupling states between economic performance and environmental pressures at different levels. It is evident that decoupling, coupling, and negative decoupling states for EG and EPI occur at the national level between 2004 and 2019. Specifically, before 2009, China’s economic development was accompanied by great environmental pressure, and although the economy grew at a high rate, the resulting environmental problems were more serious. The decoupled state of the two varies back and forth between expansive negative decoupling and strong negative decoupling, which is consistent with the continuously rising environmental pressures that China faced during that period. Despite a clear set of goals to improve energy use efficiency and reduce pollution in the 10th (2001–2005) and 11th Five-Year Plans (FYP) (2006–2010) of the Chinese government, the sloppy and inefficient developmental approach and the slow pace of transition still caused great damage to the environment. After 2010, the relationship between China’s economy and the environment exhibited a decoupled and coupled state. Specifically, during the 12th FYP period, the country’s economic boom showed a similar rate of change to the rise in environmental pressure, and the economy even grew faster than the rise in environmental pressure, indicating that the effects of the series of environmental protection measures implemented during the 12th Five-Year Plan period are beginning to emerge at this point. During the 13th FYP period, the economy and the environment remained strongly decoupled, i.e., economic growth was accompanied by a significant decrease in environmental pressure, indicating that China’s economic growth and environmental protection achieved a “win–win” situation.

#### 4.2.2. Decoupling between Environmental Pressure and Economic Performance at the Provincial level

As mentioned earlier, there are three states here, namely, C, D, and ND. Throughout the research period, these three categories changed in various ways. Each decoupling category is described in this section by the number of provinces that make up each category. According to Figure 5, the number of provinces in category D is the sum of the number of provinces belonging to the RD, SD, and WD states. Similarly, we can obtain the number of provinces in the C and ND states. Thus, we obtain the evolution of the dismissal of the three categories during the period 2004–2019, as shown in Table 4.

From Table 5, it is evident that the dominant decoupling category changed significantly between ND and D during 2010–2015, where the number of ND and D remained almost the same until 2010; however, the number of D increased significantly, and the number of ND decreased gradually during the 13th FYP. From the end of the 13th FYP, the D category dominates and the number of provinces in the ND category gradually decreases, which indicates that the state between economic growth and environmental pressure in the region gradually changes to a predominantly decoupling state.

**Figure 6:**
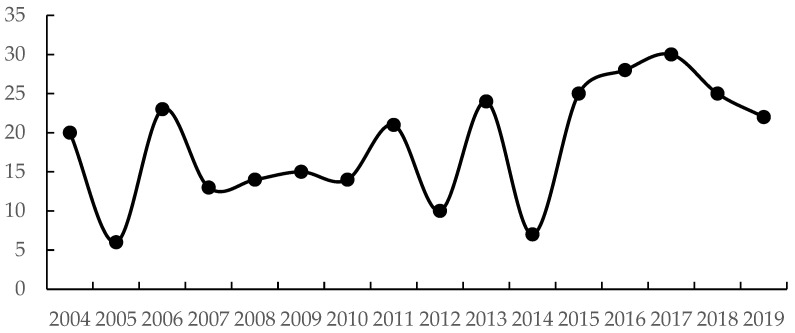
Number of provinces included in category D during the study period.

To more fully comprehend the development of the relationship between the environment and the economy, and for a detailed description of the evolution of the decoupling state in particular, we focus the further analysis on category D. The number of provinces in category D over the years is shown in Figure 6. Figure 6 shows that the number of provinces in category D fluctuates over the study period. It displays a cyclical trend from peak to trough from 2004 to 2007 and from 2011 to 2015, which stabilizes between 2007 and 2010, and shows a clear upward trend after 2015. Similarly, the number of provinces in category D during the 13th FYP is significantly higher than in the 12th, 11th, and 10th FYP. As environmental awareness rises in each region and policies are implemented, the once single-minded pursuit of economic growth is gradually changing to one that achieves maximum economic benefits at a minimal environmental cost.

**Figure 7:**
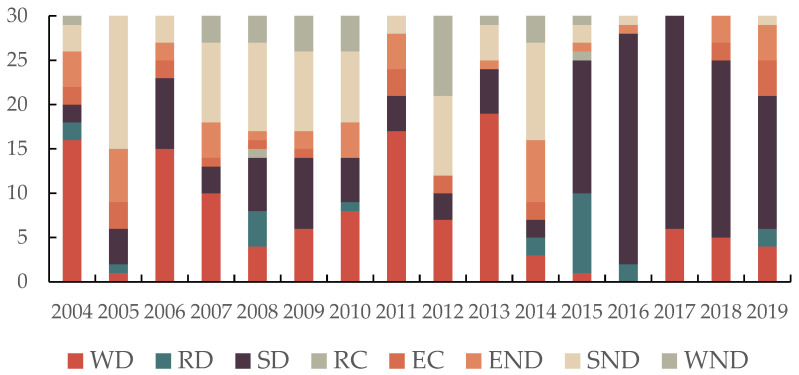
Distribution of the provinces in different decoupling status during the study period.

As mentioned earlier, the three major categories of D, C, and ND contain different subcategories such as END, SND, WND, WD, RD, SD, RC, and EC. The evolutionary patterns of these eight states have also varied during the course of the research. The development of the various states is explained by the number of provinces in each state. the number of provinces in these eight states for the period from 2004–2019 is shown in Figure 7. According to Figure 7, the decoupling status of 30 Chinese provinces, autonomous regions, and municipalities changed dynamically during 2004–2019. Among them, WD, SND, and SD were in the dominant position in different years. In 2004, 2006, 2011, and 2013, WD was in the dominant position. During this period, the economic increase was accompanied by an increase in environmental pressure, but the economic growth was faster than the increase in environmental pressure. SND was dominant in 2005, 2014, and 2007–2010. During this period, the increase in environmental pressure was evident, the economy experienced a recession, and the development of each region was in a serious state of unsustainability. However, since 2015, SD has dominated. During this period, rapid economic growth was accompanied by a significant decline in environmental pressures, and the sustainability of each region continued to increase, indicating a “win–win” situation for both economic growth and environmental protection.

**Figure 8:**
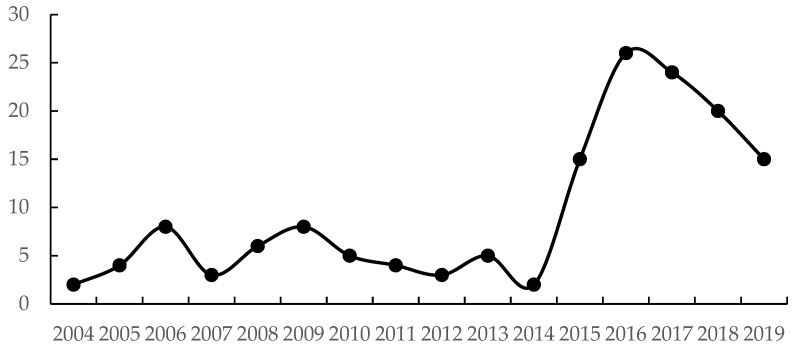
Number of provinces included in category SD during the study period.

Figure 8 shows the number of provinces where EG and EPI are in a strong decoupling state during the period 2004–2019. The fluctuation in the number curve is similar to Figure 6, i.e., the number of provinces in a SD state displays alternating peaks and valleys until 2014, when no more than a quarter of the country’s provinces were able to maintain a “win–win” situation for both economic growth and environmental protection. After 2014, the number of SD provinces rose sharply, and although it declined after 2016, more than half of the country’s provinces still showed strong decoupling, indicating that with the implementation of a series of national guidelines and strategies for environmental protection and improvement, regional environmental pressures declined significantly, and regional sustainability strengthened nationwide.

### 4.3. Analysis on the Spatial Differences of the Decoupling between Environmental Pressure and Economic Performance

We split China into four areas, namely, the eastern region, the central region, the western region, and the northeastern region, to further study the geographical disparities in the decoupling states of economic growth and environmental pressure at the provincial level in China. In addition, to describe the changes more intuitively in the number of provinces in different states over the years, in this section, we divide the study time into three parts, namely, the 11th FYP, the 12th FYP, and the 13th FYP.

Table 6 summarizes the number of provinces with different decoupling states in the four regions of China during the 11th, 12th, and 13th FYP. From the table, we found that among the four regions in China, the provinces in the coupling state are the fewest and do not show significant spatial differences. From 2005 to 2019, the number of provinces in the decoupling state in each region continued to increase, with the number of provinces in the eastern region increasing from 24 in the 11th FYP to 31 in the 13th FPYP, the number of provinces in the western region increasing from 30 to 40, and the number of provinces in the central region increasing from 17 to 24. At the same time, the number of provinces with a negative coupling status decreased significantly, for example, from 26 to 6 in the eastern region, and from 24 and 13 to 1 in the western and central regions, respectively, which indicates that the relationship between the regional economy and the environment changed significantly during this period, and the economic growth paths that create high emissions and pollution have been gradually replaced by sustainable economic growth paths.

**Figure 9:**
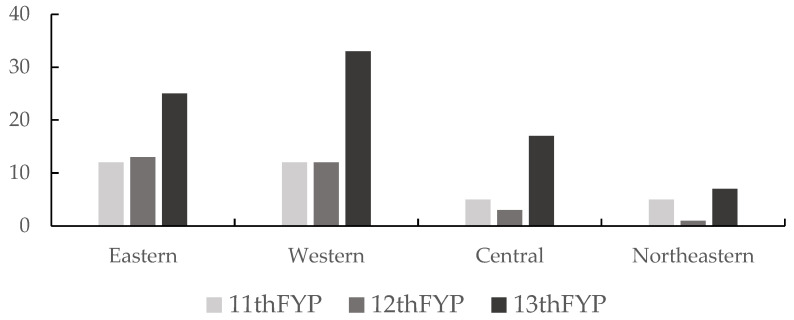
Spatial differences of the number of provinces in category SD during the study period.

In order to achieve a “win–win” scenario for economic growth and environmental protection, it is necessary to achieve both sustained environmental pressure reduction and economic growth, as we discussed in the preceding section. Therefore, we further counted the number of provinces in SD in the four major regions of China during the 11th, 12th, and 13th FPY, as shown in Figure 9. From the figure, we found that the number of provinces in SD in the eastern and western regions is basically the same during the 11th and 12th FYP periods, while the number of provinces in SD in the central and northeastern regions during the 11th FYP period is more than the 12th FYP period. In the 13th FYP period, the number of provinces with SD status in each region increased intensively, most obviously in the western region, from 12 initially to 33 currently, and in the other regions, from 12 to 25 in the eastern region, from 5 to 17 in the central region, and from 5 to 7 in the northeastern region. This shows that during the 13th FYP period, provinces nationwide achieved a reduction in economic growth and environmental pressure, and that economic development and environmental protection can achieve a “win–win” situation.

## 5. Discussion

Our findings in this study demonstrate that from 2004 to 2019, environmental preservation and economic growth successfully coexisted at the national level, strengthening the objective of sustainable regional development. Over the 16 years of China’s development, the value of nighttime lights as a proxy for economic aggregation increased by 219% and the environmental pressure index decreased from 0.481 to 0.302. Economic development places higher demands on environmental quality while providing access to new technologies. To mitigate possible environmental contamination, the central government should often enact regulatory restrictions or offer subsidies and incentives. Several studies of the environmental Kuznets curve have revealed that economic production patterns and environmental consumption patterns dominated by high inputs, high consumption, and high pollution can further undermine economic sustainability [82,83]. China’s rapid development pattern since the reform and opening up has been accompanied by inefficient resource use and increased environmental damage [84]. However, environmental behaviors such as environmental management and improvement should be viewed more as contributors to economic development rather than as obstacles [85]. Therefore, decoupling environmental impacts from economic growth can be seen not only as an opportunity for economic sustainability, but also as contributing to the goal of sustainable development. For developing countries such as China, the blind pursuit of economic prosperity without an adequate consideration of environmental and social impacts is bound to be costly [86]. As a result, the Chinese government has made environmental conservation a priority in its economic development in order to create a “win–win” situation in terms of the sustainability of both the economy and the environment.

**Figure 10:**
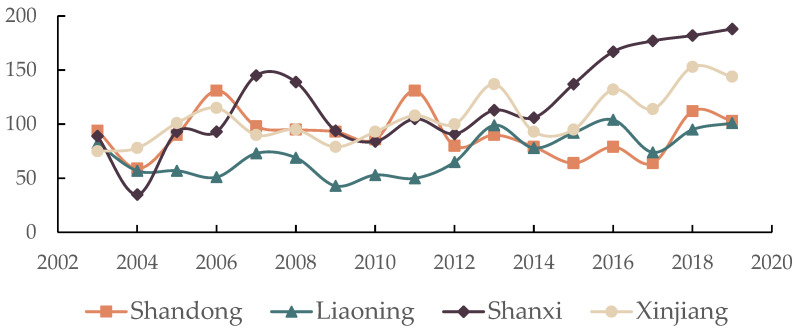
Frequency of environment-related keywords in government work reports over the years.

In fact, the decoupling of environmental impacts from economic growth is further supported by national policies and the orientation of related guidelines and strategies to address historically influenced environmental issues such as soil, air, water, and solid waste [85,87,88,89]. Table 7 summarizes the main environmental improvement pathways and sustainability projects at the national level in China, which involve agricultural and rural pollution, soil pollution prevention, water pollution prevention, and arable land quality protection. Furthermore, we selected Shandong, Liaoning, Shanxi, and Sichuan as representatives of the eastern, northeastern, central, and western regions, respectively, and used a text mining approach to mine environment-related keywords from the government work reports of each region over the years, which included environmental protection, environmental pollution, environmental co-governance, and other related terms (e.g., blue sky, white clouds, clean land, green water, and green mountains). Figure 10 shows the word frequencies of the related keywords based on text mining. We found that in the government work reports of all the years, all local government departments made mention of environment-related issues, and the frequency of environment-related keywords appearing from 2003 to 2019 increased year by year, which was especially obvious in the central and western regions. For example, environment-related keywords appeared 89 times in the government work report of Shanxi in 2003, while this value increased to 188 in 2019, and the number of appearances of environment-related keywords in the government work report of Xinjiang increased from 77 to 144. The rising frequency of environment-related keywords reflects the local government’s concern for environmental issues and the importance it attaches to them and shows the determination of local governments to prioritize environmental issues in their economic development.

The sustainability of the region depends on how the economy and the environment interact [90,91]. To sustainably reduce the issue of trade-offs between environmental preservation and economic growth, the central government must create a more effective system of environmental protection measures [84,85,86,92]. In fact, strong socioeconomic objectives such as targeted poverty alleviation, rural revitalization, dual carbon targets, and the Belt and Road Initiative are frequently used in China to achieve environmental protection. Since 2000, these objectives have resulted in an unprecedented increase in the central government’s investment in environmental sustainability across the board. China is becoming increasingly more aware of the significance and necessity of developing an “ecological civilization” and separating environmental effects from economic success. This has led to China designating sustainable development as part of its national plan. In view of the successful implementation of the national guidelines and strategies, the central government should maintain medium and long-term, large-scale investments, diversified and systematic solutions, and a coordinated and functioning organizational system with a focus on classification, which will help us to achieve further results in both economic and environmental terms. The decoupling of China’s environmental impacts from economic growth is now emerging, and Chinese evidence shows that economic growth and environmental protection can achieve a “win–win” situation.

Here, we propose several policy recommendations to sustainably improve China’s environmental impacts while maintaining economic growth. First, the central government should continue to promote the transformation of the industrial structure and increase the shift from manufacturing to service industries, while accelerating industrial restructuring to compress the structure of heavy industries with high energy consumption, high pollution, and high emissions. Second, the central government should pursue the development of clean and low-carbon energy; fiercely pursue the development of renewable energy sources such as hydropower, wind power, and solar power; broadly support energy efficient building, emission reduction, and the conservation of energy; strengthen the guiding role of scientific and technological innovation in economic development; and improve the standardization of green manufacturing and green applications. Third, the central government should promote the comprehensive conservation of energy resources and improve the comprehensive prevention and control of solid waste such as tailings, metallurgical slag, iron and steel scrap, and waste non-ferrous metals. Fourth, the central government should pay more attention to environmental performance when evaluating the political promotion of local officials and reverse the dominance of economic performance in political propaganda under the leadership of local officials.

## 6. Conclusions

This study adopts NTL data as a proxy for regional economic development and constructs a comprehensive quantification system of environmental pressure covering 6 primary and 11 secondary indicators. The following are the study’s primary conclusions: Firstly, NTL data as a proxy for economic performance represent a valid source for assessing the decoupling status. Second, from 2004 to 2019, China’s environmental conditions improved, and environmental pressure decreased. Third, the empirical results show that the relationship between the economy and the environment in China continues to improve, with a “win–win” situation for economic growth and environmental protection at the national level. At the provincial level, most provinces have decoupled economic growth from environmental impacts, but this state is unstable and environmental pressures are still likely to increase.

Although our findings are interesting, there are limitations: firstly, the analysis has not yet been extended to a comparative analysis of performance between cities; secondly, there are inevitable imprecisions in the nighttime lighting used as a proxy for economic performance, on the one hand, there are differences in data acquisition, and on the other hand, despite the variety of data correction and analysis methods, there is still no universally accepted data processing method that can serve as a standard. In future work, economic growth and environmental impacts can be evaluated at small scales based on NTL data, and factors leading to different decoupling states can be investigated using, for example, the Logarithmic Mean Division Index (LMDI) method, so that more targeted recommendations can be made to improve the relationship between the economy and the environment, thus achieving a “win–win” situation for both economic growth and environmental protection.

## Figures and Tables

**Table 1:** The main findings of the study on the relationship between economy and environment and their authors.

No.	Relationships between Environment and Economy	Authors
1	Inverted-U shape (EKC hypothesis in long-term)	Grossman and Krueger [33], Hao and Liu et al. [43], Zhu et al. [39], AL-Mulali et al. [44]
	Inverted-U shape (EKC hypothesis in short-term)	Khalid Ahmed and Wei Long [31], Zhao et al. [34]
2	Rejection of the EKC hypothesis	Hettige et al. [45], Ozturk and Al-Mulali [46], Perman and Stern [47], Wang et al. [48]
3	A monotonic relationship	Stern and Common [49], Azomahou et al. [50], Jaunky [51]
4	N-shaped	Yan-Qing Kang et al. [35], Yoonseok Lee et al. [36], Hannes Egli and Thomas M. Steger [37], O. Zaim and F. Taskin [38], Zhu et al. [39]
5	Special relationships (linear positive relationship at the stage of low income and then plateaus)	Bertinelli and Strobl [52], Ahmed et al. [53]
6	U-shaped	Lopez and Litra [32]

**Table 2:** Environmental pressure index (EPI) related indicators.

First-Level Indicator	Secondary Indicator	Description	Units
Air quality	PM_2.5_ concentration	Annual average of PM_2.5_ concentration	Μg/m^3^
Greenhouse gas	Carbon dioxide emissions	CO_2_ emissions per capita	Ton
Waste gas	Sulfur dioxide emissions	SO_2_ emissions per capita	Ton
	Nitrogen oxide emissions	NO_x_ emissions per capita	Ton
	Smoke and dust emissions	Smoke and dust emissions per capita	Ton
Wastewater	Industrial wastewater discharge	Industrial wastewater discharge per capita	Ton
	Chemical oxygen demand (COD)	Chemical oxygen demand per capita	Ton
Solid waste	Hazardous waste	Hazardous waste generation per capita	Ton
	General industrial solid waste	General industrial solid waste generation per capita	Ton
Environmental Monetization	Regional environmental infrastructure development investment	Regional environmental infrastructure construction investment per capita	CNY
	Regional industrial pollution control completed investment	Per capita regional industrial pollution control completed investment	CNY

**Table 3:** NTL data value of sample province from 2004 to 2019 (Unit: DN).

Province	2004	2007	2010	2013	2016	2019
Anhui	85,063	130,330	133,212	398,374	441,794	666,974
Beijing	241,992	341,549	292,066	293,819	346,528	426,005
Fujian	126,262	139,552	120,968	413,085	423,163	595,036
Gansu	66,047	66,173	90,882	150,642	167,993	217,499
Guangdong	847,102	793,558	521,234	107,9492	105,2835	145,5367
Guangxi	60,822	63,545	54,758	206,772	251,557	324,731
Guizhou	30,686	26,106	26,498	184,091	245,172	294,047
Hainan	23,425	17,828	25,575	75,458	86,228	107,918
Hebei	258,737	298,117	311,181	448,416	494,570	738,914
Henan	167,072	218,124	205,669	454,382	487,109	790,192

**Table 4:** The result of ∆*EPI*, ∆*EG*, and *λ* for the sample city Beijing.

	2004	2005	2006	2007	2008	2009	2010	2011
∆*EPI*	0.2631	−0.2030	0.0945	−0.0199	−0.0974	0.0841	−0.0885	0.0024
∆*EG*	−0.0297	0.3892	0.0909	−0.0687	−0.022	−0.0665	−0.0623	0.3566
λ	−8.83	−0.52	1.04	0.29	4.24	−1.26	1.42	0.01
	**2012**	**2013**	**2014**	**2015**	**2016**	**2017**	**2018**	**2019**
∆*EPI*	0.0828	0.0871	0.1192	−0.1405	0.1792	−0.0460	−0.1877	0.1086
∆*EG*	−0.0865	−0.1881	0.0295	0.0186	0.1245	0.0408	0.0273	0.1496
λ	−0.96	−0.46	4.03	−7.53	1.44	−1.13	−6.87	0.73

**Table 5:** Evolution of the number of provinces in three decoupling categories.

Year	C	D	ND
2004	2	20	8
2005	3	6	21
2006	2	23	5
2007	1	13	16
2008	2	14	14
2009	1	15	14
2010	0	14	16
2011	3	21	6
2012	2	10	18
2013	0	24	6
2014	2	7	21
2015	1	25	4
2016	0	28	2
2017	0	30	0
2018	2	25	3
2019	3	22	5

**Table 6:** Spatial differences of the number of provinces in three decoupling categories.

	Eastern			Western			Central			Northeastern		
	C	D	ND	C	D	ND	C	D	ND	C	D	ND
11th FYP	1	24	26	2	30	21	1	17	13	1	9	5
12th FYP	1	28	16	6	31	17	1	20	15	0	7	8
13th FYP	0	31	6	2	40	1	3	24	1	0	10	2

**Table 7:** Some of the environmental sustainability projects that are being implemented or have been completed in China.

	Name	Governing Agencies
Program 1	Carbon neutral and carbon peaking strategy	State Council
Program 2	Agricultural and Rural Pollution Control Action Plan	Ministry of Ecology and Environment Ministry of Agriculture and Rural Affairs
Program 3	The Three-year Action Plan of the Blue-Sky Defense War	Ministry of Ecology and Environment
Program 4	Soil Pollution Prevention and Control Action Plan	Ministry of Ecology and Environment
Program 5	Water Pollution Control Action Plan	Ministry of Ecology and Environment
Program 6	Arable Land Quality Protection and Enhancement Project	Ministry of Agriculture and Rural Affairs
Program 7	Air Pollution Control Action Plan	Ministry of Ecology and Environment
Program 8	Grassland Ecological protection subsidy incentive projects	Ministry of Agriculture and Rural Affairs and Rural Affairs Ministry of Finance
Program 9	Beijing-Tianjin Sandstorm Source Control Project	National Forestry and Grassland Administration
Program 10	Central Financial Forest Ecological Benefit Compensation Fund Project	National Forestry and Grassland Administration Ministry of Finance
Program 11	Reforestation Project	National Forestry and Grassland Administration
Program 12	Natural Forest Protection Project	National Forestry and Grassland Administration

## Data Availability

Not applicable.
